# Simultaneous Inhibition of PGE_2_ and PGI_2_ Signals Is Necessary to Suppress Hyperalgesia in Rat Inflammatory Pain Models

**DOI:** 10.1155/2016/9847840

**Published:** 2016-07-13

**Authors:** Ryusuke Sugita, Harumi Kuwabara, Kazufumi Kubota, Kotaro Sugimoto, Toshihiro Kiho, Atsushi Tengeiji, Katsuhiro Kawakami, Kohei Shimada

**Affiliations:** ^1^Cardiovascular-Metabolics Research Laboratories, Daiichi Sankyo Co., Ltd., Tokyo 140-8710, Japan; ^2^Frontier Research Laboratories, Daiichi Sankyo Co., Ltd., Tokyo 140-8710, Japan; ^3^Biological Research Laboratories, Daiichi Sankyo Co., Ltd., Tokyo 140-8710, Japan; ^4^Medicinal Chemistry Research Laboratories, Daiichi Sankyo Co., Ltd., Tokyo 140-8710, Japan; ^5^Venture Science Laboratories, Daiichi Sankyo Co., Ltd., Tokyo 140-8710, Japan; ^6^Global Project Management Department, Daiichi Sankyo Co., Ltd., Tokyo 140-8710, Japan

## Abstract

Prostaglandin E_2_ (PGE_2_) is well known as a mediator of inflammatory symptoms such as fever, arthritis, and inflammatory pain. In the present study, we evaluated the analgesic effect of our selective PGE_2_ synthesis inhibitor, compound I, 2-methyl-2-[*cis*-4-([1-(6-methyl-3-phenylquinolin-2-yl)piperidin-4-yl]carbonyl amino)cyclohexyl] propanoic acid, in rat yeast-induced acute and adjuvant-induced chronic inflammatory pain models. Although this compound suppressed the synthesis of PGE_2_ selectively, no analgesic effect was shown in both inflammatory pain models. Prostacyclin (PGI_2_) also plays crucial roles in inflammatory pain, so we evaluated the involvement of PGI_2_ signaling in rat inflammatory pain models using prostacyclin receptor (IP) antagonist, RO3244019. RO3244019 showed no analgesic effect in inflammatory pain models, but concomitant administration of compound I and RO3244019 showed analgesic effects comparable to celecoxib, a specific cyclooxygenase- (COX-) 2 inhibitor. Furthermore, coadministration of PGE_2_ receptor 4 (EP4) antagonist, CJ-023423, and RO3244019 also showed an analgesic effect. These findings suggest that both PGE_2_ signaling, especially through the EP4 receptor, and PGI_2_ signaling play critical roles in inflammatory pain and concurrent inhibition of both signals is important for suppression of inflammatory hyperalgesia.

## 1. Introduction

Nonsteroidal anti-inflammatory drugs (NSAIDs), including aspirin, indomethacin, diclofenac, and ibuprofen, are widely used in the treatment of inflammatory symptoms such as fever, arthritis, and inflammatory pain. These drugs reduce the production of prostaglandins by blocking both COX-1 and COX-2. COX-1 is constitutively expressed in various tissues and plays a role in homeostatic functions such as gastric epithelial cytoprotection and renal blood flow maintenance. Thus, traditional NSAIDs cause side effects to the gastrointestinal (GI) tract and renal function [1]. As a result, COX-2 selective inhibitors such as celecoxib, rofecoxib, and valdecoxib were developed. Although COX-2 inhibitors show an analgesic effect and fewer adverse effects on the GI tract, a cardiovascular hazard in patients with long-term use of these compounds has been reported. Eventually, all such compounds except for celecoxib were withdrawn from the market [2, 3].

Prostaglandins are synthesized through conversion of arachidonic acid to an unstable intermediate, prostaglandin H_2_ (PGH_2_), by COX-1 and COX-2. PGH_2_ is further catalyzed to PGE_2_, prostacyclin (PGI_2_), prostaglandin F_2*α*_, and thromboxane A_2_ (TXA_2_) by PGE synthase (PGES), PGI synthase, prostaglandin F synthase, and thromboxane A synthase, respectively. Traditional NSAIDs suppress all prostanoids' synthesis by inhibiting both COXs. On the other hand, COX-2 inhibitors selectively suppress COX-2-derived prostanoids which are mainly induced by inflammatory stimuli. Among all prostanoids, PGE_2_ is the most common prostanoid produced by a variety of cells and tissues and plays a pivotal role in inflammatory responses. There are three different PGES, cytosolic PGES (cPGES) and two microsomal PGES (mPGES-1 and mPGES-2). cPGES and mPGES-2 are constitutive enzymes, whereas mPGES-1 is induced by inflammatory stimuli [4–6]. Furthermore, many reports suggest that mPGES-1 plays an important role in inflammatory symptoms [7–9]; thus mPGES-1 is thought to be a therapeutic target alternative to traditional NSAIDs and COX-2 inhibitors. Presently, some mPGES-1 inhibitors have been discovered and the pharmacological profiles have been reported [10–12], suggesting the therapeutic potential of pharmacological inhibition of this enzyme for relieving inflammatory responses. On the other hand, PGI_2_ also plays an important role in inflammation and inflammatory pain. IP receptor-deficient mice showed a reduced writhing response in an acetic acid-induced acute inflammatory pain model [13, 14]. In addition, IP antagonists also showed anti-inflammatory and analgesic effects in rodent arthritis models [15, 16]. Thus, the IP receptor is thought to be another interesting target for anti-inflammatory and analgesic drug development. At this time, it remains inconclusive which action is a more promising target for an analgesic drug.

Recently, we have discovered quinoline derivatives which are orally available and have a potent inhibitory effect on PGE_2_ synthesis in rats. We have revealed that these compounds are highly selective for PGE_2_ synthesis inhibition [17, 18], and we have previously reported the antipyretic and anti-inflammatory profile of compound A in a rat LPS-induced pyrexia and adjuvant-induced arthritis model [17]. In the present study, we evaluated analgesic effects of another quinoline derivative, compound I, in acute and chronic inflammatory pain models. In addition, we investigated the function of PGE_2_ and PGI_2_ signaling in these models using our compound and an IP antagonist. Among all PGE_2_ receptors, EP4 plays an important role in inflammatory pain [19]; thus an EP4 antagonist was used in the present pain model.

## 2. Material and Methods

### 2.1. Animals

All experimental procedures were performed in accordance with the in-house guidelines of the Institutional Animal Care and Use Committee of Daiichi Sankyo Co., Ltd. For the adjuvant-induced inflammatory pain model, male Lewis rats were purchased at the age of 5 weeks to 6 weeks from Charles River Japan Inc. (Kanagawa, Japan). For the yeast-induced inflammatory pain model, male Wistar-Imamichi rats were purchased at the age of 4 weeks to 5 weeks from the Institute for Animal Reproduction (Ibaraki, Japan). For the rat macrophage assay, male Sprague-Dawley rats were purchased at the age of 8 weeks from Japan SLC Inc. (Shizuoka, Japan). The animals were housed in an air-conditioned room with controlled temperature (23 ± 2°C) and humidity (55 ± 20%) and a 12 h light/dark cycle (light from 8:00 to 20:00). They were fed and given water* ad libitum* throughout the experimental period unless otherwise noted. They were acclimated for about 1 week before use.

### 2.2. Test Compounds

**Figure 1:**
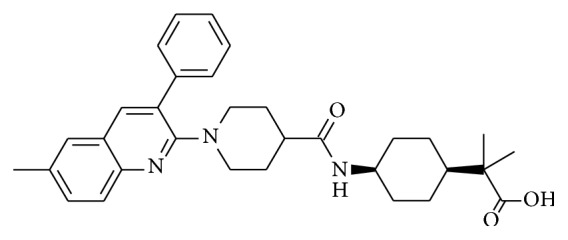
Chemical structure of compound I.

Compound I (2-methyl-2-[*cis*-4-([1-(6-methyl-3-phenylquinolin-2-yl)piperidin-4-yl]carbonyl amino)cyclohexyl] propanoic acid) was synthesized at Medicinal Chemistry Research Laboratories, Daiichi Sankyo Co., Ltd. Detailed information of its chemical synthesis is described in the patent [18]. The chemical structure is shown in Figure 1. IP antagonist, RO3244019, (*R*)-3-phenyl-2-(5-phenyl-benzofuran-2-ylmethoxycarbonylamino)-propionic acid, and EP4 antagonist, CJ-023423,* N*-[*N*-[2-[4-(2-ethyl-4,6-dimethyl-1*H*-imidazo[4,5-*c*]pyridin-1-yl)phenyl]ethyl]carbamoyl]-4-methylbenzenesulfonamide, were synthesized at Medicinal Chemistry Research Laboratories, Daiichi Sankyo Co., Ltd. Celecoxib (4-[5-(4-methylphenyl)-3-(trifluoromethyl)pyrazol-1-yl]benzenesulfonamide) was purchased from Cayman Chemical Company (Ann Arbor, MI). All compounds were suspended in 0.5% methyl cellulose 400 solution and sterilized (Wako Pure Chemical Industries, Ltd., Osaka, Japan) and a volume of 5 mL/kg was administered orally to animals.

### 2.3. Rat Macrophage Assay

Rat peritoneal macrophages were prepared as described previously [20]. Briefly, 5% (w/v) peptone (BD Biosciences, Franklin Lakes, NJ) and 5% (w/v) starch (BD Biosciences) solution was intraperitoneally injected into male Sprague-Dawley rats at a dose of 5 mL/100 g body weight. Four days later, the rats were euthanized by cutting the carotid artery under anesthesia, and the peritoneal macrophages were collected. Macrophages were seeded in a 96-well plate at a density of 200,000 cells/well and pretreated with 100 *μ*M aspirin (Sigma-Aldrich) at 37°C for 2 h in RPMI 1640 (Life Technologies) supplemented with 10% FBS and penicillin-streptomycin liquid (Life Technologies). The cells were washed twice to remove nonadherent cells and then preincubated at 37°C for 1 h with a compound or vehicle in 200 *μ*L of RPMI medium. The cells were treated with 10 *μ*g/mL lipopolysaccharide (LPS, lot number 0111:B4, Sigma-Aldrich) at 37°C and 5% CO_2_ for 24 h, and PGE_2_, 6-keto PGF_1*α*_ (stable metabolite of PGI_2_), PGF_2*α*_, and TXB_2_ (stable metabolite of TXA_2_) in the medium were measured by EIA (Assay Designs). Each experiment was performed in duplicate. The results of LPS(−), LPS(+), and celecoxib (positive control) were the same as our published paper [17] because this experiment was performed in the same plate.

### 2.4. Adjuvant-Induced Chronic Inflammatory Pain Model

Experiments were performed according to the method previously described [21] with slight modifications. Briefly, an adjuvant was prepared by suspending heat-killed dried* M. butyricum* (lot number 7047934, BD Biosciences) in dry-sterilized liquid paraffin (Wako Pure Chemical Industries Ltd.) and then sonicating with a Sonifier Cell Disruptor 200 (Branson Ultrasonic, Danbury, CT). The adjuvant (100 *μ*g* M. butyricum*/0.05 mL/paw) was injected intradermally into the heels of the right hind footpads of male Lewis rats. Rats were fasted overnight on Day 18, and the pain response of the animals was examined by gently flexing the tarsotibial joint of the uninjected foot 5 times at intervals of 2 s to 3 s on Day 19. Animals squeaking at every flexion were defined as pain-positive. The pain-positive rats were then randomly divided into groups and test compounds or the vehicle was orally administered (*n* = 6). The pain response was examined at 1, 2, 3, and 4 h after administration by flexing the uninjected foot 5 times in the same way, and the number of instances of squeaking was recorded as the pain score. The score of noninjected animals was 0 all the time in this experiment. After the final measurement, animals were euthanized and the left (uninjected) hind paws were immediately removed and weighed. The paws were snap frozen with liquid nitrogen and stored at −80°C until use. The paws were then crushed with a Cryo-Press® (Microtec Co., Ltd., Chiba, Japan) and homogenized with a Polytron® homogenizer at 4°C for 60 s four times the volume of each paw of phosphate buffered saline (PBS) with 10 mM EDTA and 100 *μ*M indomethacin. The homogenate was centrifuged at 1,560 ×g for 15 min at 4°C, and 1 mL of the supernatant fraction was further centrifuged at 9,500 ×g for 5 min at 4°C. The supernatant was then stored at −20°C until the measurement of prostanoid contents. Paws of noninjected animals were used as a control. Prostanoids were measured with respective EIA kit (Cayman Chemicals) according to the manufacturer's instructions.

### 2.5. Yeast-Induced Acute Inflammatory Pain Model

Experiments were performed according to the method previously described with slight modification [22]. Briefly, male Wistar-Imamichi rats weighing from 60 g to 80 g were fasted overnight and 0.1 mL of 20% suspension of dead Brewer's yeast (Sigma) was injected intradermally into their right hind footpads. A hypernociceptive stimulation was produced by applying 250 g of pressure to the inflamed paw twice at 16 h and 18 h after yeast injection by a balance pressure device (Ugo-Basile, Italy). A constant increase in pressure was applied to the inflamed paw 18.5 h after the yeast injection and the pain threshold was determined by measuring the pressure in g at the time when the animal began to cry. Animals with a pain threshold between 60 g and 120 g were selected for assays and test compounds or vehicle was immediately administered to them orally (*n* = 6). Then the pain threshold at 1, 2, 3, and 4 h after dosing was measured. After the final measurement, the animals were euthanized and inflamed paws were collected. The supernatants of homogenized paw samples were prepared as described above. About 2 cm of each spinal cord including the lumbar segment was also harvested and supernatants were prepared as described above. Prostanoids were measured with a respective EIA kit (Cayman Chemical) according to the manufacturer's instructions.

### 2.6. Statistical Analysis

In a macrophage assay, data are expressed as the mean ± SD and other data are expressed as the mean ± SEM. In* in vitro* experiments, IC_50_ values were derived from four point titrations. In the inflamed tissue assay, the percent inhibition of prostanoid content by a compound was calculated by the following equation: 1 −  {(prostanoid content of a compound treated animal) − (a prostanoid content of a normal group)} ÷ {(prostanoid content of a vehicle-treated group) − (prostanoid content of a normal group)}   × 100. ID_50_ values were calculated based on linear regression lines obtained from the percent inhibitions and the logarithmic values of the doses by the least squares method. The statistical analysis for the prostanoid content was performed by Dunnett's test, otherwise by Steel's test for multiple comparisons.

## 3. Results

### 3.1. Compound I Suppresses PGE_2_ Production Selectively in Rat Macrophages

**Figure 2:**
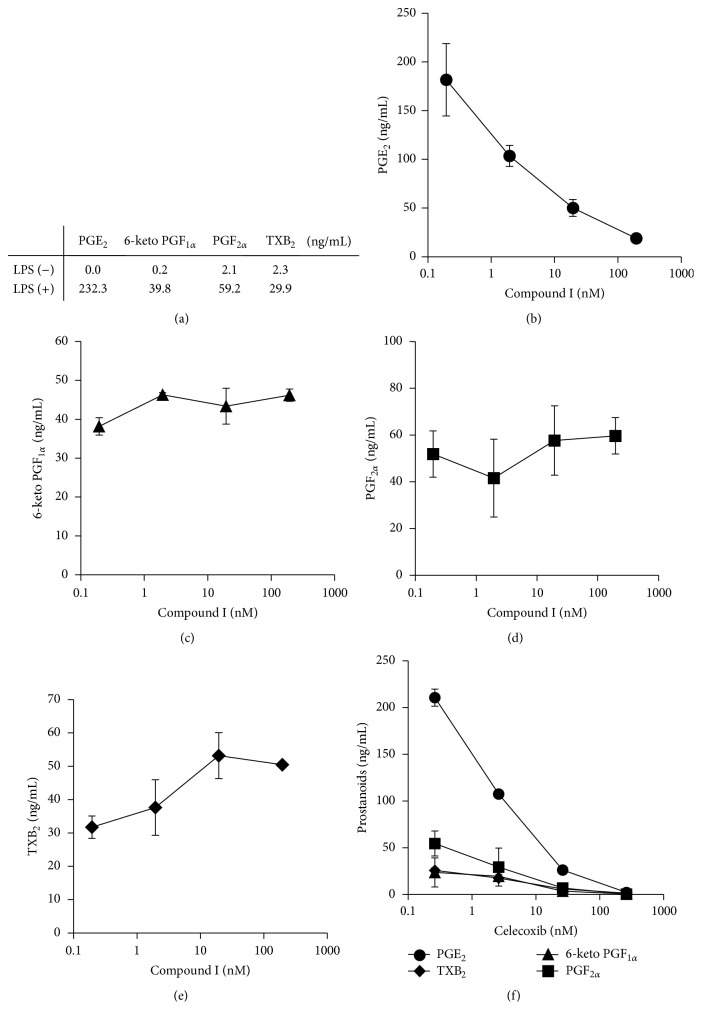
Induction of prostanoids synthesis and inhibitory profile of compound A in rat peritoneal macrophages. (a) The production of PGE_2_, 6-keto PGF_1*α*_, PGF_2*α*_, and TXB_2_ with or without LPS treatment. (b) to (e) Dose-dependent effects of compound A on the production of PGE_2_ (b), 6-keto PGF_1*α*_ (c), PGF_2*α*_ (d), and TXB_2_ (e) in rat macrophages. (f) Dose-dependent effects of celecoxib on the production of PGE_2_, 6-keto PGF_1*α*_, PGF_2*α*_, and TXB_2_ in rat macrophages. Results are shown as the mean ± SD (in duplicate). A representative result from reproduced experiments is shown.

To elucidate the inhibitory profile of our compound, production of prostaglandins was evaluated in a rat macrophage assay system. In this assay, LPS stimulation induced the production of PGE_2_, 6-keto PGF_1*α*_, PGF_2*α*_, and TXB_2_. Among them, the amount of PGE_2_ was the highest (Figure 2(a)). Compound I suppressed the production of PGE_2_ in a dose-dependent manner and its IC_50_ was 1.9 nM (Figure 2(b)). The production of 6-keto PGF_1*α*_ and PGF_2*α*_ was not suppressed (Figures 2(c) and 2(d)), whereas TXB_2_ production was accelerated (Figure 2(e)). Celecoxib suppressed all kinds of prostanoid synthesis (Figure 2(f)).

### 3.2. Compound I Selectively Reduces PGE_2_ Production but Shows No Analgesic Effect in Adjuvant-Induced Chronic Inflammatory Pain Models

**Figure 3:**
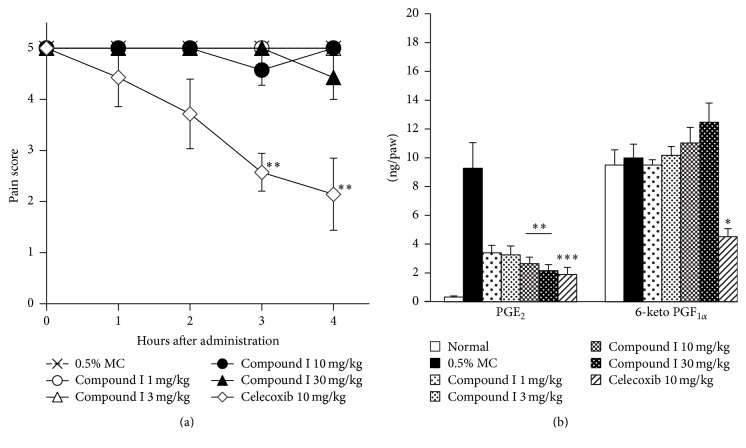
Analgesic effect of compound I and celecoxib in adjuvant-induced chronic inflammatory pain in rats. (a) Time course of pain score. Male Lewis rats received a single, right hind-paw intradermal injection of* M. butyricum* (100 *μ*g/0.05 mL/paw) in a liquid paraffin emulsion on Day 0. At Day 19, animals with established hyperalgesia (pain score = 5) were orally administered compounds or 0.5% MC. Pain response was examined 1, 2, 3, and 4 h after administration. (b) Effect of compound I and celecoxib on inflamed tissue prostanoids' production in adjuvant-induced chronic inflammatory pain model. Inflamed paw was obtained after the last measurement of pain score. Results are shown as the mean ± SEM (*n* = 6/group). The statistical analysis was performed by Steel's test and Dunnett's test for pain score (a) and prostanoids' content (b), respectively. ^*∗*^ *p* < 0.05; ^*∗∗*^ *p* < 0.01; ^*∗∗∗*^ *p* < 0.001 for compound treated versus 0.5% MC (0 mg/kg) treated animals.

In order to investigate the analgesic effect of compound I, an adjuvant-induced chronic inflammatory pain model was used. Chronic pain was well-established 19 days after adjuvant injection and the pain continued throughout the measurement. While celecoxib (10 mg/kg) reduced the pain score from 5 ± 0 to 2.1 ± 0.7 at 4 h after dosing, compound I showed little or no effect (Figure 3(a)). After the last measurement, paw tissues were harvested and the content of PGE_2_ and 6-keto PGF_1*α*_ was measured. PGE_2_ production was increased from 0.3 ± 0.08 ng/paw (noninjected animal) to 9.3 ± 1.8 ng/paw by adjuvant injection, whereas production of 6-keto PGF_1*α*_ was almost unchanged (from 9.5 ± 1.1 ng/paw to 10.0 ± 1.0 ng/paw). Compound I selectively reduced PGE_2_ production with an ID_50_ value of less than 1 mg/kg (Figure 3(b)), whereas celecoxib reduced both PGE_2_ and 6-keto PGF_1*α*_ in inflamed tissue. Inhibition activity of PGE_2_ was almost the same level between 30 mg/kg of compound I and 10 mg/kg of celecoxib (Figure 3(b)).

### 3.3. Compound I Shows No Analgesic Effect in Yeast-Induced Acute Inflammatory Pain Models

**Figure 4:**
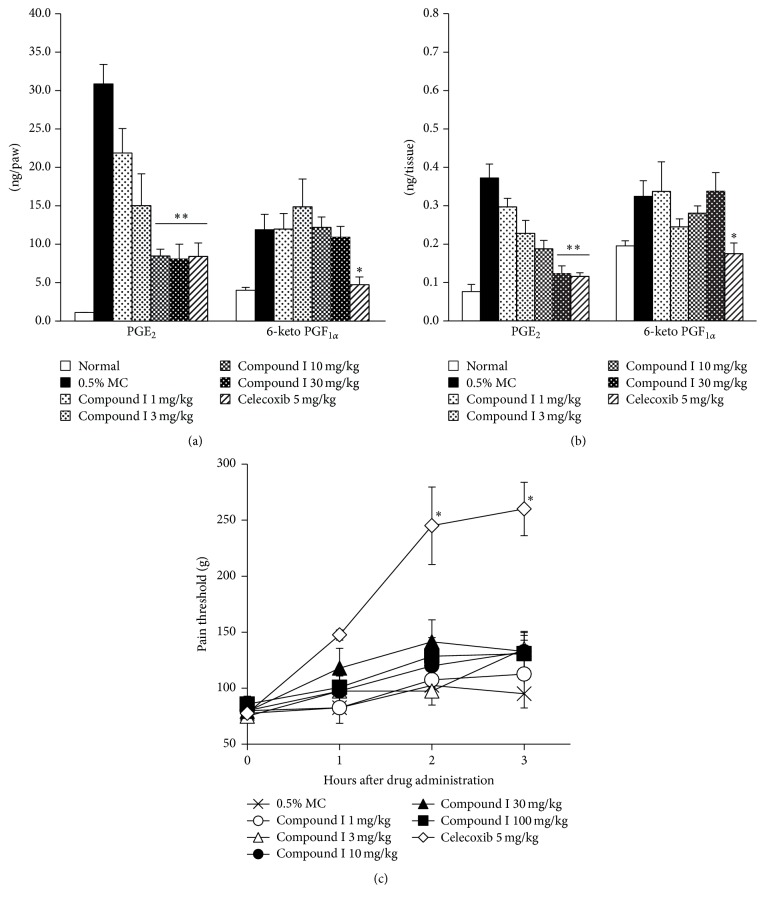
Analgesic effect of compound I and celecoxib in yeast-induced acute inflammatory pain in rats. (a) Effect of compound I and celecoxib on inflamed paw prostanoids' production in yeast-induced acute inflammatory pain model. Inflamed paw was obtained after the last measurement of pain threshold. (b) Effect of compound I and celecoxib on lumbar area of spinal cord prostanoid production in yeast-induced acute inflammatory pain model. Spinal cord was obtained after the last measurement of pain threshold. (c) Time course of pain threshold. Male Wistar-Imamichi rats received a single, right hind-paw intradermal injection of dead Brewer's yeast (20%/0.1 mL/paw). After 18.5 h, compounds or 0.5% MC were administered orally to animals with established hyperalgesia (pain threshold: 60~120 g). Pain response was examined 1, 2, 3, and 4 h after administration. Results are shown as the mean ± SEM (*n* = 6/group). The statistical analysis was performed by Dunnett's test and Steel's test for prostanoids' content ((a) and (b)) and pain threshold (c), respectively. ^*∗*^ *p* < 0.05; ^*∗∗*^ *p* < 0.01 for compound treated versus 0.5% MC (0 mg/kg) treated animals.

A yeast-induced acute inflammatory pain model is frequently used for evaluation of analgesic effect of NSAIDs, so we used this model to assess the analgesic effect of our compound. After the injection of yeast, production of PGE_2_ and 6-keto PGF_1*α*_ was increased in both the inflamed paw (from 1.1 ± 0.02 ng/paw to 30.9 ± 2.6 ng/paw and from 4.0 ± 0.4 ng/paw to 11.9 ± 2.0 ng/paw, resp.) and spinal cord (from 0.08 ± 0.02 ng/tissue to 0.37 ± 0.04 ng/tissue and from 0.2 ± 0.02 ng/tissue to 0.32 ± 0.04 ng/tissue, resp.). Compound I selectively reduced PGE_2_ synthesis in a dose-dependent manner in both inflamed paw and spinal cord with an ID_50_ value of 2.9 mg/kg and 4.2 mg/kg, respectively (Figures 4(a) and 4(b)). However, this compound showed no analgesic effect (Figure 4(c)). On the other hand, 5 mg/kg of celecoxib reduced both PGE_2_ and 6-keto PGF_1*α*_ in inflamed paw and spinal cord (Figures 4(a) and 4(b)) and showed an analgesic effect 2 h and 3 h after administration (Figure 4(c)). Pain threshold of noninjected animal is about 300 g in this model (data not shown), and so celecoxib improved inflammatory hyperalgesia almost to normal level.

### 3.4. Concomitant Administration of Compound I and IP Antagonist Showed Analgesic Effect

**Figure 5:**
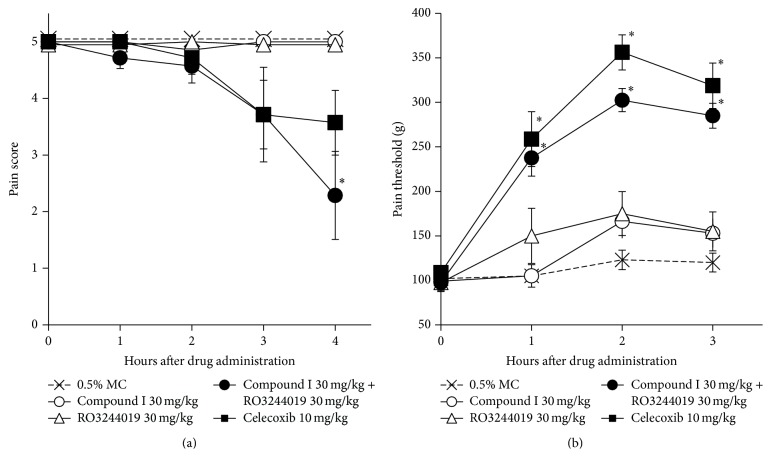
Analgesic effect of compound I and/or RO3244019 in acute and chronic inflammatory pain models. (a) Results of adjuvant-induced chronic inflammatory pain model. Male Lewis rats received a single, right hind-paw intradermal injection of* M. butyricum* (100 *μ*g/0.05 mL/paw) in a liquid paraffin emulsion on Day 0. At Day 19, animals with established hyperalgesia (pain score = 5) were orally administered compounds or 0.5% MC. Pain response was examined 1, 2, 3, and 4 h after administration. (b) Male Wistar-Imamichi rats received a single, right hind-paw intradermal injection of dead Brewer's yeast (20%/0.1 mL/paw). After 18.5 h, compounds or 0.5% MC were administered orally to animals with established hyperalgesia (pain threshold: 60 g–120 g). Pain threshold was measured 1, 2, 3, and 4 h after administration. Results are shown as the mean ± SEM (*n* = 6/group). The statistical analysis was performed by Steel's test for multiple comparisons. ^*∗*^ *p* < 0.05 for compound treated versus 0.5% MC (0 mg/kg) treated animals.

To identify the reason why our compound did not relieve inflammatory pain, we investigated whether other prostanoids contribute to the present pain models. Since PGI_2_ is also reported to play a role in inflammatory pain, we tested the analgesic effect of an IP antagonist RO3244019 in the inflammatory pain models. In the adjuvant-induced pain model, RO3244019 showed no analgesic effect. On the other hand, concomitant administration of compound I and RO3244019 showed an equivalent analgesic effect to celecoxib (Figure 5(a)). Furthermore, in the yeast-induced inflammatory pain model, concomitant administration of these two compounds improved the pain threshold at a level comparable to celecoxib, whereas RO3244019 or compound I alone showed no analgesic effect (Figure 5(b)).

### 3.5. Concomitant Administration of EP4 Antagonist and IP Antagonist Also Showed Analgesic Effect in Adjuvant-Induced Inflammatory Pain Model

**Figure 6:**
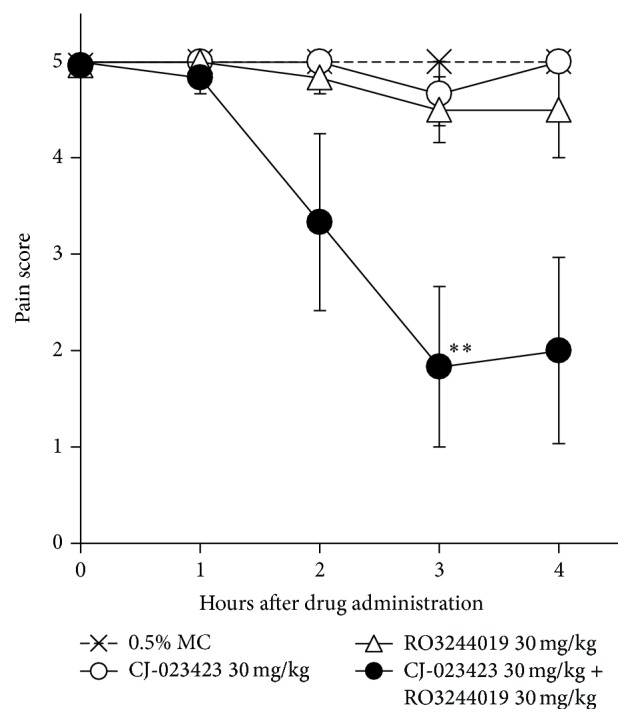
Analgesic effect of CJ-023423 and/or RO3244019 in adjuvant-induced chronic inflammatory pain model. Male Lewis rats received a single, right hind-paw intradermal injection of* M. butyricum* (100 *μ*g/0.05 mL/paw) in a liquid paraffin emulsion on Day 0. At Day 19, animals with established hyperalgesia (pain score = 5) were orally administered compounds or 0.5% MC. Pain response was examined 1, 2, 3, and 4 h after administration. Results are shown as the mean ± SEM (*n* = 6/group). The statistical analysis was performed by Steel's test for multiple comparisons. ^*∗∗*^ *p* < 0.01 for compound treated versus 0.5% MC (0 mg/kg) treated animals.

Among PGE_2_ receptors, EP4 is reported to play an important role in inflammatory pain [19]. Thus, we evaluated the effect of EP4 antagonist CJ-023423 in an adjuvant-induced inflammatory pain model. As shown in Figure 6, EP4 antagonist and IP antagonist showed little or no effect on the pain scores, whereas concomitant administration of these two compounds significantly improved them.

## 4. Discussion

PGE_2_ plays a critical role in inflammatory symptoms such as fever, edema, or inflammatory pain. Traditional NSAIDs or COX-2 inhibitors are widely used to treat these symptoms. We previously reported that a novel selective PGE_2_ synthesis inhibitor shows antipyretic and anti-inflammatory effects [17]. In the present study, we evaluated the analgesic potential of our compound in inflammatory pain models.

In a rat macrophage assay, compound I selectively and strongly inhibited LPS-induced PGE_2_ production. On the other hand, this compound did not change the production of 6-keto PGF_1*α*_ and PGF_2*α*_ and enhanced the production of TXB_2_. This may be the result of the redirection of PGH_2_ metabolism from PGE_2_ to TXB_2_. Furthermore, mPGES-1 is critically involved in the synthesis of PGE_2_ induced by LPS [23]. Taken together, compound I probably inhibits mPGES-1. This inhibitory profile was similar to that of another of our compounds as reported previously [17]. Other papers reported that mPGES-1 deficiency or a pharmacological inhibition of mPGES-1 also shows the prostanoids' redirection [24–27]. However, the inhibitory profile against each prostanoid differs among them [28], suggesting that reactivity may differ depending on the expression profile of terminal synthases in target cells or tissues.

Previous reports advocate that the inhibition of PGE_2_ synthesis is necessary to relieve the inflammatory hyperalgesia [7, 8, 10, 12]. Thus, we investigated whether our compound can relieve hyperalgesia in rat inflammatory pain models which were well-established in evaluating traditional NSAIDs and COX-2 inhibitors. In an adjuvant-induced chronic pain model, adjuvant injection induced the production of PGE_2_. Compound I suppressed the production of PGE_2_, whereas 6-keto PGF_1*α*_ synthesis was not significantly changed and rather tended to be increased at a high dose. The maximum dose (30 mg/kg) of compound I suppressed PGE_2_ synthesis to almost the same degree as celecoxib. However, celecoxib, but not our compound, showed an analgesic effect in this model. Unlike compound I, celecoxib also suppressed 6-keto PGF_1*α*_ synthesis, suggesting that only the inhibition of PGE_2_ is insufficient to relieve inflammatory hyperalgesia. In a yeast-induced acute inflammatory pain model, PGE_2_ and 6-keto PGF_1*α*_ production in inflamed paw were increased. Because intraspinal PGE_2_ has been shown to act on central sensitization in an acute arthritis model [29], we measured prostaglandins' production in spinal cord tissue. In this model, PGE_2_ and 6-keto PGF_1*α*_ content were increased in the spinal cord. Compound I suppressed PGE_2_ production selectively in both inflamed paw and spinal cord, and the maximum dose of compound I suppressed PGE_2_ production almost to the same degree as celecoxib. However, while celecoxib showed a strong analgesic effect, compound I showed no effect. Celecoxib suppressed both PGE_2_ and 6-keto PGF_1*α*_ in both tissues, further suggesting the contribution of other prostanoid signals to inflammatory pain.

Among prostanoids, PGI_2_ is also known as a mediator of inflammation and inflammatory pain. To assess the involvement of PGI_2_ signaling in inflammatory pain models, the IP antagonist, RO3244019, was used as reported previously [30]. In the adjuvant-induced chronic inflammatory pain model, single administration of RO3244019 showed no analgesic effect. On the other hand, concomitant administration of compound I and RO3244019 improved the pain score as well as or more than celecoxib, and the same result was obtained in the yeast-induced acute inflammatory pain model. These results strongly suggest that both PGE_2_ and PGI_2_ signals are sufficient to induce inflammatory hyperalgesia and inhibition of only one prostaglandin signal is insufficient to relieve the pain. In the adjuvant-induced inflammatory pain model, the amount of 6-keto PGF_1*α*_ was not increased by adjuvant injection, indicating that normally existing PGI_2_ also acts as a mediator of inflammatory pain. Although the exact mechanism remains to be clarified, the adjuvant injection may increase IP expression followed by accelerating IP signal in this model. Thus, PGI_2_ signal may contribute to inflammatory pain only in inflammatory condition. Another study demonstrated that pain behavior is not reduced in mPGES-1 knockout mice in a zymosan-evoked hyperalgesia model, and it is suggested that other prostanoids may compensate the loss of PGE_2_ synthase in mPGES-1-deficient mice [31]. In the present study, the same compensation may occur in PGE_2_ selectively suppressed mice by compound I. On the other hand, not only PGE_2_ but also other prostaglandins contribute to peripheral and central sensitization. For example, intrathecal injection of prostaglandin D_2_ induced hyperalgesia [32]. Although the precise mechanism is still unknown, PGF_2*α*_ also induces allodynia [33]. Although our results suggest that the inhibition of PGE_2_ and PGI_2_ synthesis is enough to improve inflammatory hyperalgesia, further investigation for the involvement of other prostanoids in inflammatory pain may be needed.

We next assessed which of the EP receptors is important to inflammatory pain. Among all EP receptors, EP4 is predominantly induced in DRG neurons by adjuvant injection and mainly contributes to inflammatory pain hypersensitivity [19]. In addition, blockade of the EP4 receptor shows anti-inflammatory and analgesic effects in inflammation models [34, 35]. Thus, EP4 antagonist CJ-023423, which was reported to show high selectivity for the EP4 receptor and reverse inflammation and inflammatory pain [35, 36], was used to assess the contribution of this subtype to our inflammatory pain model. However, single treatment of CJ-023423 did not improve the pain score in the present adjuvant-induced inflammatory pain model. In the previous paper, a carrageenan-induced mechanical hyperalgesia model and adjuvant-induced weight bearing deficit model were used as acute and chronic inflammatory pain models, respectively [35]. They performed the test 2 days after adjuvant injection when arthritis was not established yet, whereas we measured the pain score 19 days after adjuvant injection when arthritis was fully established. Because EP4 expression is induced in DRG neuron from 2 to 7 days after adjuvant injection [19], EP4 contributes to inflammation and inflammatory pain at that period and EP4 antagonist shows analgesic effect [35]. However, in a later phase when arthritis is fully established, it is uncertain whether EP4 still mainly contributes to inflammation. In the present study, coadministration of CJ-023423 and RO3244019 reversed adjuvant-induced inflammatory pain, suggesting that not only the EP4 but also the IP signal contributes to inflammatory hyperalgesia in such a late phase. Although contributions of other EP receptors were not investigated, this result suggests that the EP4 receptor dominantly contributes to the PGE_2_ signal in the chronic inflammatory pain model.

In summary, we evaluated the analgesic effect of a PGE_2_ selective inhibitor, but it showed no effect in rat acute and chronic inflammatory pain models. On the other hand, dual inhibition of PGE_2_ and PGI_2_ signals relieved the pain behavior at a level comparable to COX-2 inhibition, suggesting that both PGE_2_ and PGI_2_ signals play important roles in inflammatory pain. Among PGE_2_ receptors, the EP4 receptor dominantly contributes to PGE_2_ signaling during inflammatory hyperalgesia.

## Abbreviations

COX: Cyclooxygenase
DMEM: Dulbecco's modified Eagle's medium
DMSO: Dimethyl sulfoxide
EIA: Enzyme immunoassay
EP: Prostaglandin E_2_ receptor
FBS: Fetal bovine serum
GI: Gastrointestinal
IP: Prostaglandin I_2_ receptor
mPGES-1: Microsomal prostaglandin E synthase-1
LPS: Lipopolysaccharide
NSAIDs: Nonsteroidal anti-inflammatory drugs
PGE_2_: Prostaglandin E_2_
PGF_2*α*_: Prostaglandin F_2*α*_
PGH_2_: Prostaglandin H_2_
PGI_2_: Prostaglandin I_2_
6-keto PGF_1*α*_: 6-keto prostaglandin F_1*α*_
TXA_2_: Thromboxane A_2_
TXB_2_: Thromboxane B_2_.
